# Juvenile hormone promotes paracellular transport of yolk proteins via remodeling zonula adherens at tricellular junctions in the follicular epithelium

**DOI:** 10.1371/journal.pgen.1010292

**Published:** 2022-06-27

**Authors:** Hongyuan Zheng, Ningbo Wang, Jiaqi Yun, Huijing Xu, Jiebing Yang, Shutang Zhou

**Affiliations:** State Key Laboratory of Cotton Biology, School of Life Sciences, College of Agriculture, Henan University, Kaifeng, Henan, China; Friday Harbor Laboratories: University of Washington, UNITED STATES

## Abstract

Juvenile hormone (JH) acts as a gonadotrophic hormone stimulating insect vitellogenesis and oogenesis. Paracellular transport of yolk proteins through intercellular channels (patency) in the follicular epithelium is a developmentally regulated and evolutionarily conserved process during vitellogenesis. However, the mechanisms underlying patency opening are poorly understood. Using the migratory locust *Locusta migratoria* as a model system, we report here that JH-regulated remodeling of zonula adherens (ZA), the belt-like adherens junction maintaining physical linking between follicle cells controlled the opening of patency. JH triggered phosphorylation of Partitioning defective protein 3 (Par3) via a signaling cascade including G protein-coupled receptor (GPCR), small GTPase Cell division cycle 42 (Cdc42) and atypical Protein kinase C (aPKC). Par3 phosphorylation resulted in its disassociation from β-Catenin, the cytoplasmic partner of ZA core component E-Cadherin. Release of Par3 from the β-Catenin/E-Cadherin complex caused ZA disassembly at tricellular contacts, consequently leading to patency enlargement. This study provides new insight into how JH stimulates insect vitellogenesis and egg production via inducing the opening of paracellular route for vitellogenin transport crossing the follicular epithelium barrier.

## Author summary

Vitellogenesis is one of the most emblematic processes in female reproduction of oviparous animals. In many insects, the yolk protein precursor, vitellogenin (Vg) is synthesized in the fat body and transported to oocytes through the intercellular spaces (patency) among follicular cells. Juvenile hormone (JH), the arthropod-specific sesquiterpenoid plays a crucial role in paracellular Vg transport, but the molecular mechanisms of JH-stimulated patency remain elusive. In the present study, we show that JH acts via the GPCR-Cdc42-aPKC signaling cascade that triggers the phosphorylation of Par3, a critical scaffold protein of zonula adherens. JH-dependent Par3 phosphorylation results in its dissociation from the β-Catenin/E-Cadherin complex, consequently leading to patency opening for Vg transport. The findings reveal an important mechanism by which JH induces the remodeling of zonula adherens for the opening of paracellular route for Vg transport crossing the follicular epithelium barrier in the ovary.

## Introduction

Vitellogenesis is crucial to egg development and embryonic growth after oviposition in oviparous animals. In insects, vitellogenesis is characterized by vitellogenin (Vg) synthesis in the fat body (equivalent to vertebrate liver and adipose tissue), secretion into the hemolymph, transport through intercellular channels (a condition known as patency) in the follicular epithelium and uptake by maturing oocytes [1,2]. Juvenile hormone (JH), a sesquiterpenoid produced in corpora allata is the primary hormone governing vitellogenesis in a variety of insect species [3–5]. Cumulative studies have established that JH acts via its receptor complex comprised of Methoprene-tolerant (Met) and Taiman (Tai) in activating the transcription of JH-responsive genes, consequently achieving the previtellogenic effect on fat body competency and the vitellogenic effect on Vg synthesis [4–9]. In the mosquito *Aedes aegypti*, JH triggers Met phosphorylation for enhanced transcriptional activity via a signaling cascade including RTK, PLC, IP3, CaMKII and PKC, which promotes the previtellogenic development [10,11]. Moreover, JH promotes the phosphorylation of Serine/Arginine-rich (pre-mRNA) Splicing Factor (SRSF) via the RTK-PI3K-Akt pathway, thereby inducing the generation of Tai A/B isoforms that potentiates the transcriptional activity of ecdysone receptor (EcR) for blood meal-induced vitellogenesis in *A*. *aegypti* [12]. In *Drosophila melanogaster*, JH acts via the RTK-PLC-PKC cascade for phosphorylation of ultraspiracle (USP), and thus potentiates 20-hydroxyecdysone (20E) action [13]. In the cotton bollworm *Helicoverpa armigera*, JH induces Met phosphorylation for enhanced dimerization with Tai and transcriptional activity in the expression of JH-early response gene, *Kr-h1* [14]. In the migratory locust *Locusta migratoria*, JH triggers FoxO dephosphorylation via a signaling cascade including Leucine carboxyl methyltransferase 1 (LCMT1) and PP2A for fat body cell polyploidization and massive Vg synthesis [15]. JH also promotes Kr-h1 phosphorylation and recruits the coactivator, CREB-binding protein, consequently stimulating the expression of *Ribosomal protein L36* and Vg synthesis in the fat body of *L*. *migratoria* [16]. In addition, JH activates the GPCR-PLC-PKCι cascade for Vg receptor (VgR) phosphorylation that is prerequisite for VgR-mediated endocytosis of Vg and subsequent translocation of VgR from oocyte cytoplasm to membrane [17]. While extensive studies have been conducted for elucidating JH-induced Vg synthesis in the fat body, the molecular and cellular mechanisms of ovarian Vg transportation are poorly understood.

One of vitellogenic events in the follicle epithelium is patency opening. The follicular epithelium has barrier function during oocyte maturation. Hemolymph yolk protein precursors must pass the follicular epithelium via patency to reach oocyte membrane for subsequent VgR-mediated endocytosis [1,2]. JH-dependent patency has been described in insects belonging to diverse orders, from the primitive species with panoistic ovary like *L*. *migratoria* to the more advanced ones with meroistic ovaries like *A*. *aegypti* [2,5,18,19]. The involvement of Na^+^/K^+^-ATPase in patency induction has been demonstrated in several insect species including the kissing bug *Rhodnius prolixus*, the beetle *Tenebrio molitor*, the moth *Heliothis virescens* and *L*. *migratoria* [20–23]. In vitellogenic females of *L*. *migratoria*, JH triggers phosphorylation of Na^+^/K^+^-ATPase α-subunit via a signaling cascade including GPCR, RTK, PLC, IP3R and PKC, thereby activating Na^+^/K^+^-ATPase and inducing patency for Vg transportation [24]. In addition to Na^+^/K^+^-ATPase, an early study on *R*. *prolixus* vitellogenesis has suggested a link between zonula adherens (ZA) disassembly and patency enlargement in tricellular vertices [25]. In *L*. *migratoria*, ZA disassembly occurs concurrently with patency dilatation during vitellogenic phase [26]. A recent report has demonstrated that patency is regulated by dynamic remodeling of adhesion and actomyosin contractility at cell vertices during *D*. *melanogaster* oogenesis [27]. ZA is a belt-like adherens junction encircling epithelial cells to form mechanical adhesion between neighboring cells and building up a compact epithelial sheet. The core components of ZA consist of a single-pass transmembrane protein, E-cadherin (E-Cad) and its cytoplasmic partner, β-Catenin (β-Cat) [28,29]. In *D*. *melanogaster*, depletion of *E-Cad* leads to enlarged patency in the follicular epithelium at mid-vitellogenic stage [27,30]. While extracellular portion of E-Cad mediates trans-interactions between neighboring cells, its intracellular domain associating with β-Cat anchors to actin cytoskeleton for stable cell-cell adhesions [28,31]. Partitioning defective protein 3 (Par3), a critical scaffold protein of ZA binds to β-Cat and phosphoinositide membrane lipids, which cooperates with actin filaments and stabilizes junction structures [32–37]. Atypical PKC (aPKC) and Partitioning defective protein 6 (Par6) constitute a classical Par complex with Par3 [38,39]. aPKC is activated by Par6 via pesudosubstrate displacement, consequently leading to Par3 phosphorylation and dissociation from the partner proteins [40,41]. Despite the description of ZA and cell junction in patency dynamics, little is known about the mechanisms underlying JH-dependent patency.

The migratory locust *L*. *migratoria*, bearing the panoistic ovary and synchronously matured oocytes, has been a long-standing model for studying JH-stimulated vitellogenesis and female reproduction [4,5]. In *L*. *migratoria*, JH controls Vg synthesis in the fat body, secretion into the hemolymph, transport through patency in the follicular epithelium and uptake by the maturing oocytes [2,3]. With this model system, we found that ZA core protein β-Cat retreated from tricellular junctions but remained at bicellular contacts when JH-induced patency enlarged. JH triggered Par3 phosphorylation via a signaling cascade including GPCR, Cell division cycle 42 (Cdc42), Par6 and aPKC. Phosphorylated Par3 disassociated from β-Cat, thereby leading to ZA disassembly and patency dilatation.

## Results

### Remodeling of zonula adherens contributes to JH-induced patency

**Fig 1:**
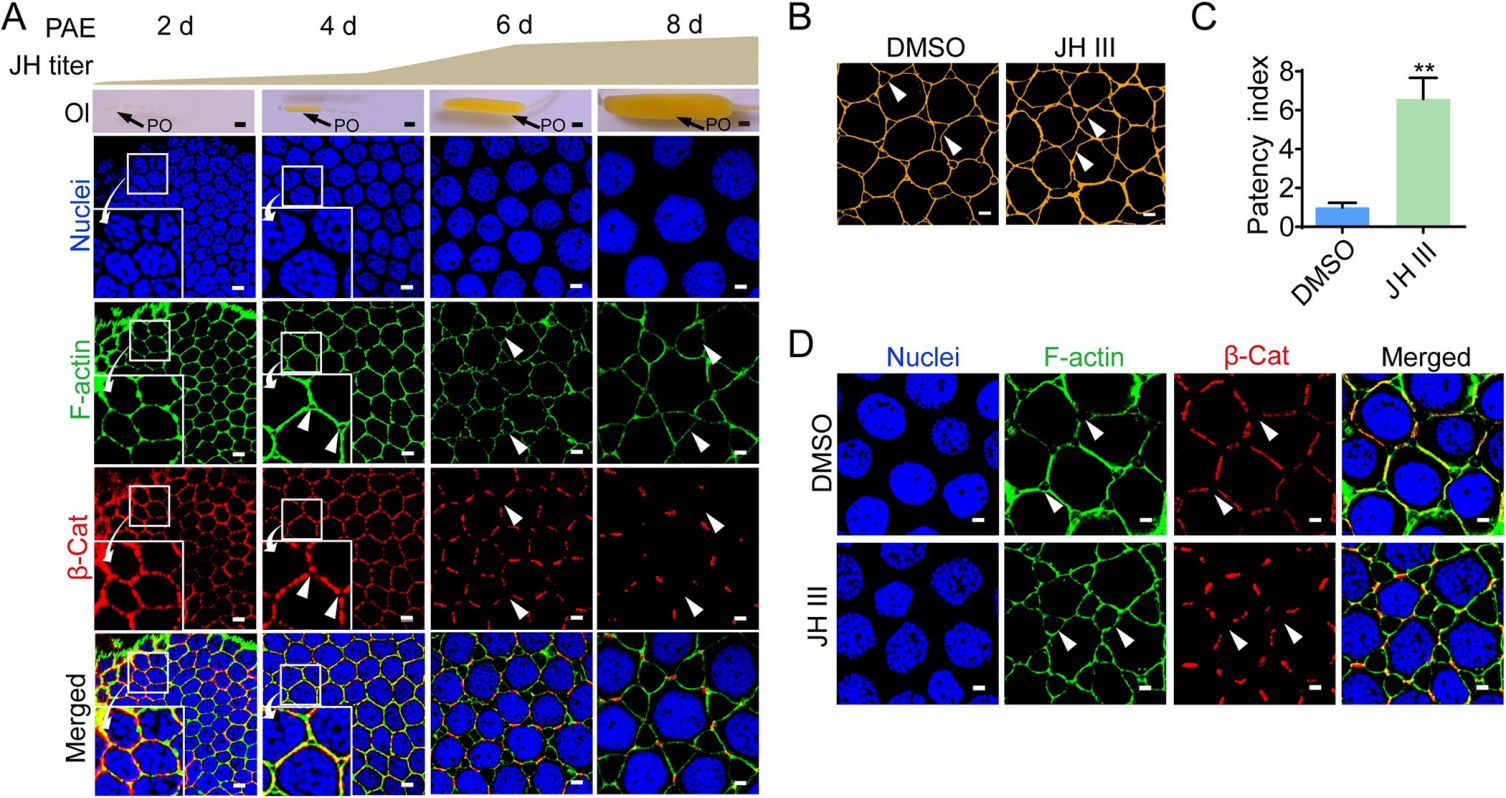
Dynamics of β-Cat distribution along with patency in the follicular epithelium. (A) Subcellular localization of β-Cat in the follicular epithelium of adult females during the first gonadotropic cycle. PAE, post adult eclosion; Arrow heads indicate patency. Large square is the amplification of small square. Ol, ovariole; PO, primary oocyte. Scale bars: ovariole, 0.5 mm; follicular epithelium, 5 μm. (B) Patency in cultured follicular epithelia of 7-day-old adult females treated by JH at 0.1 μM for 1 h. Scale bars: 5 μm. (C) Statistical analysis of patency index in cultured follicular epithelium of 7-day-old adult females. **, *P* < 0.01 when compared to the DMSO control. n = 8–10. (D) Subcellular localization of β-Cat in cultured follicle epithelium of 7-day-old adult females under JH treatment. Arrow heads indicate patency. Scale bars: 5 μm.

The first gonadotrophic cycle of adult female locusts under this study was about 8 days, and vitellogenesis started at approximately 4 days post adult eclosion (PAE). To investigate the dynamics of ZA structure in the follicular epithelium of adult females during the first gonadotropic cycle, we performed immunostaining showing the intercellular localization of β-Cat, a marker of ZA structure. In ovarian follicular epithelia of 2-day-old previtellogenic females in which no patency was observed, β-Cat distributed at both tricellular contacts and bicellular junctions (Fig 1A). At 4 days PAE when patency started opening, β-Cat retreated from tricellular vertices but sustained in bicellular junctions (Fig 1A). When patency enlarged at 6–8 days PAE, less staining of β-Cat was observed at bicellular junctions (Fig 1A). The observations indicate that ZA remodeling is involved in patency induction among follicular cells of vitellogenic females. In *L*. *migratoria*, hemolymph JH is undetectable at adult female eclosion, progressively elevates in previtellogenic stage, sharply increases in early vitellogenic phase and rises to a peak in late vitellogenic stage (Fig 1A) [42]. Reduced β-Cat staining along with patency dilatation suggest a link between ZA disassembly and increased JH titers. As patency is induced by JH during locust vitellogenesis [24] and enlarged to the maximum size on day 8 (Fig 1A), we next cultured the ovarian follicular epithelium of 7-day-old adult females to further examine the responsiveness of β-Cat distribution to JH treatment. As shown in Fig 1B, application of JH led to enlarged patency (Fig 1B) in the cultured follicular epithelium. Statistically, the patency index of JH-treated follicular epithelium increased 6.6 folds compared to the DMSO control (Fig 1C). Upon JH treatment, β-Cat distribution shrank and limited to bicellular junctions (Fig 1D). The observations suggest the induction of JH on ZA disassembly and patency opening.

**Fig 2:**
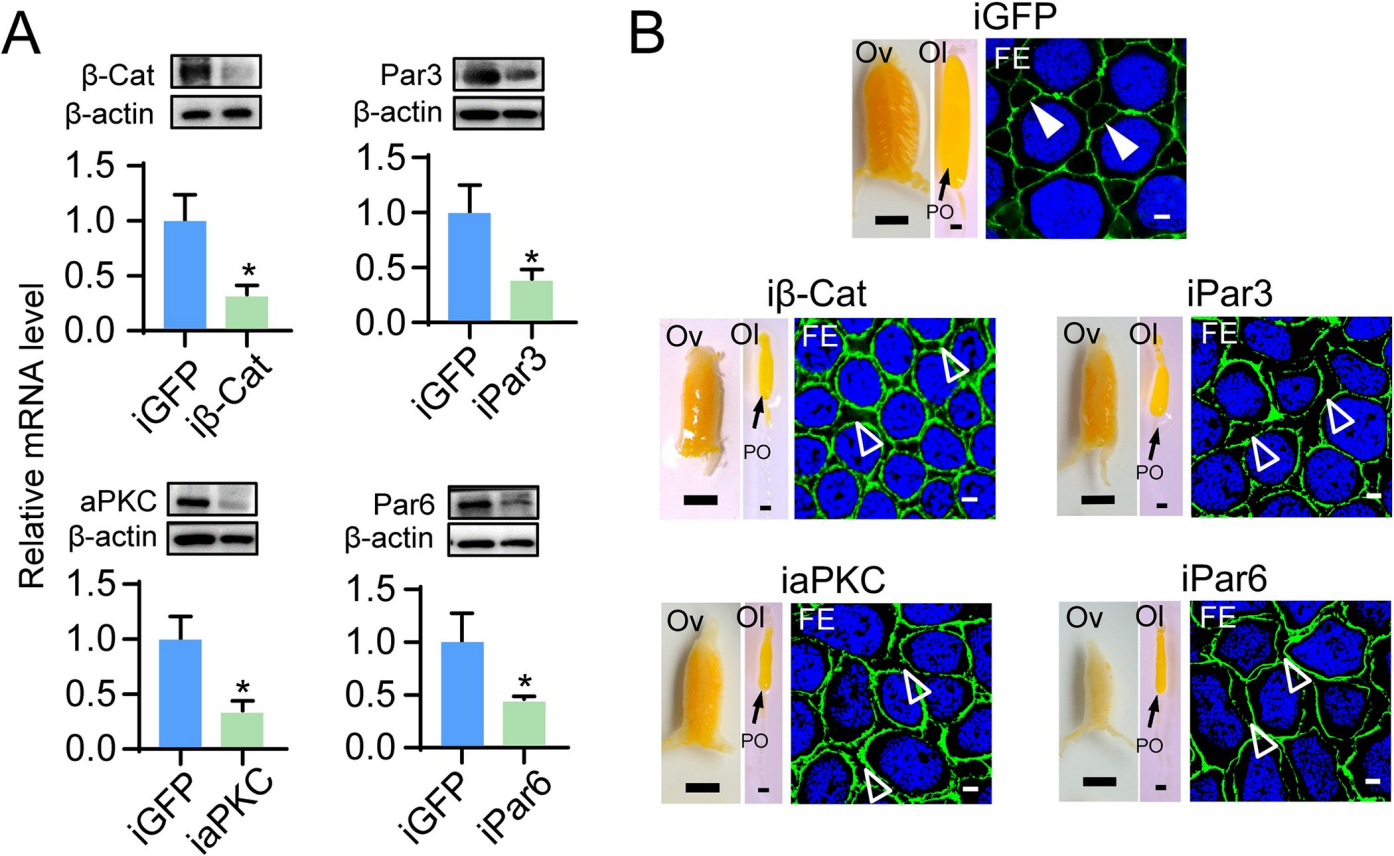
Effect of *β-Cat*, *Par3*, *aPKC* and *Par6* knockdown on patency and vitellogenesis. (A) qRT-PCR and Western blot showing RNAi-mediated knockdown efficiency of *β-Cat* (iβ-Cat), *Par3* (iPar3), *aPKC* (iaPKC) and *Par6* (iPar6) in the ovary of adult females at 8 days PAE. *, *P* < 0.05. n = 6–8. (B) Representative phenotypes of follicular epithelium (FE), primary oocytes (Po), ovarioles (Ol) and ovaries (Ov) of adult females subjected to *β-Cat*, *Par3*, *aPKC* or *Par6* RNAi. Arrow heads indicate patency. Empty arrow heads indicate break of bicellular junctions. Scale bars: ovary, 5 mm; ovariole, 0.5 mm; follicular epithelium, 5 μm.

As the role of β-Cat in JH-dependent locust vitellogenesis had not been previously determined by gene knockdown, we conducted *β-Cat* (GenBank: MT036387) RNAi in vitellogenic adult female locusts. qRT-PCR showed that injection of *β-Cat* dsRNA (iβ-Cat) caused 67% reduction of its mRNA levels in the ovary compared to the dsGFP control (Fig 2A). Western blot demonstrated a remarkable reduction of β-Cat protein levels in the ovary of *β-Cat*-depleted adult females compared to the dsGFP control (Figs 2A and S1). Knockdown of *β-Cat* resulted in dissociation of neighboring follicle cells and disappearance of typical patency, accompanied by impaired oocyte maturation and arrested ovarian growth (Fig 2B). The results indicate that bicellular adherens junction and ZA integrity are critical for maintenance of monolayer follicular epithelium and intercellular patency. As Par complex plays an important role in ZA hemostasis, we performed dsRNA-mediated RNAi of *Par3* (GenBank: MT036384), *aPKC* (GenBank: MT036383) and *Par6* (GenBank: MT036385) to elucidate their role in locust vitellogenesis. qRT-PCR showed that 60.5%, 63.1% and 54% knockdown efficiencies were obtained with RNAi of *Par3* (iPar3), *aPKC* (iaPKC) and *Par6* (iPar6), respectively (Fig 2A). Likewise, the protein levels of Par3, aPKC and Par6 markedly decreased as demonstrated by Western blots (Figs 2A and S1). *Par3* knockdown also resulted in abnormal follicular epithelium and loss of patency as well as blocked oocyte maturation and inhibited ovarian growth (Fig 2B). Defective phenotypes of follicular epithelia, patency, oocytes and ovaries were also observed with depletion of *aPKC* and *Par6* (Fig 2B), resembling those seen with *β-Cat* and *Par3* knockdown. Notably, knockdown of *β-Cat*, *Par3*, *aPKC* or *Par6* appeared to result in complete dissociation of neighboring follicle cells, abnormal follicular epithelium and loss of patency. The results suggest that loss of *β-Cat*, *Par3*, *aPKC* or *Par6* function could cause breakdown of follicle cell adhesions and patency development. Collectively, these observations indicate a crucial role of Par complex in the integrity of follicular epithelia, vitellogenesis and oogenesis.

### JH induces aPKC and Par3 phosphorylation for ZA disassembly and patency opening

**Fig 3:**
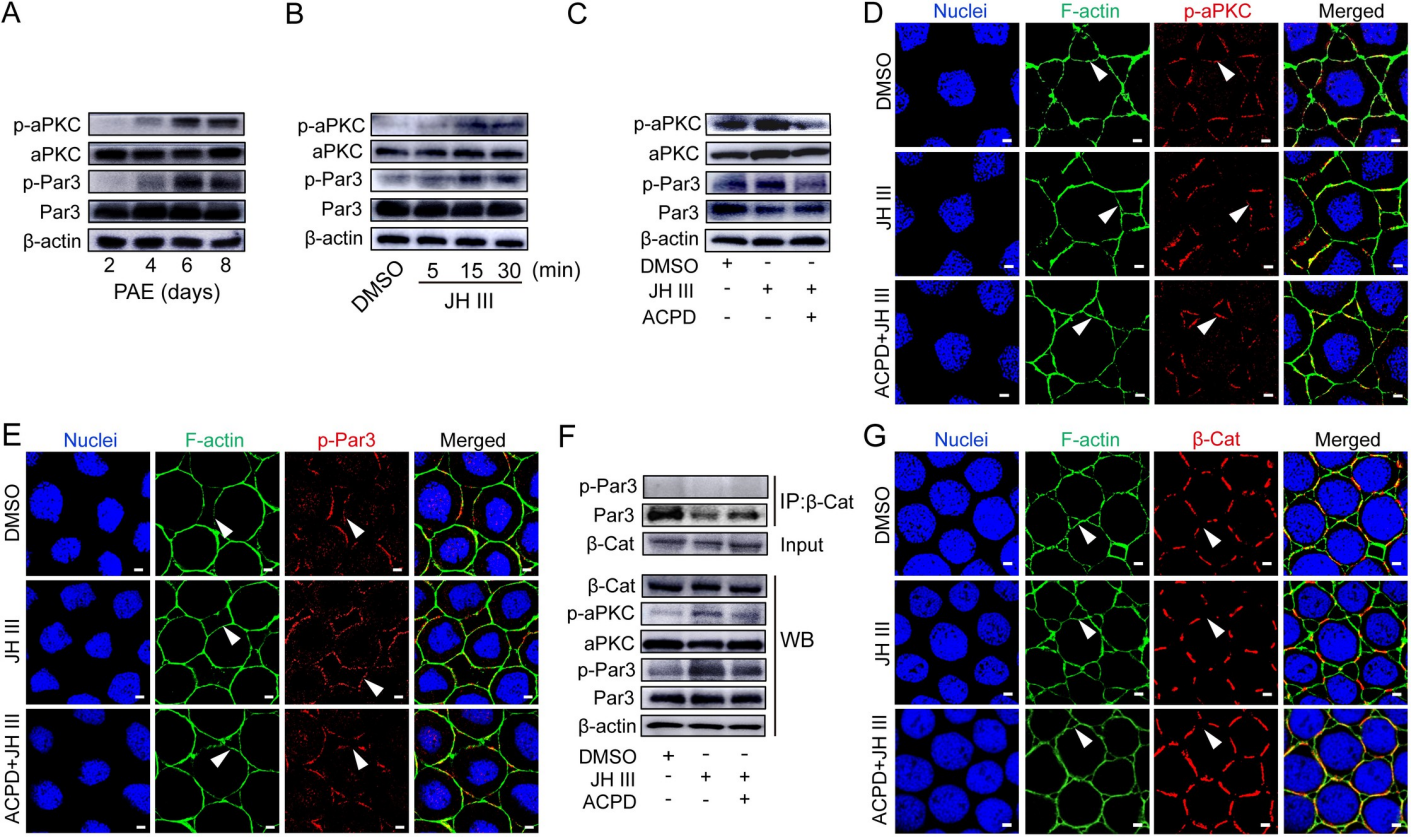
JH-induced phosphorylation of aPKC and Par3 as well as zonula adherens disassembly. (A) The levels of phosphorylated aPKC (p-aPKC) and Par3 (p-Par3) in the follicular epithelium of adult females during the first gonadotropic cycle. (B) Phosphorylation of aPKC and Par3 in cultured follicular epithelia of 7-day-old females treated by JH at 0.1 μM for 5–30 min. (C) Suppression of ACPD on JH-induced aPKC and Par3 phosphorylation in cultured follicle cells of 7-day-old females. ACPD was applied at 0.1 μM prior to JH treatment. (D) Distribution of p-aPKC in cultured follicular epithelium from adult females on day 8 and treated with DMSO, JH and ACPD+JH, respectively. Blue: nuclei. Green: F-actin. Arrow heads indicate patency. Scale bars: 5 μm. (E) Distribution of p-Par3 in cultured follicular epithelia from adult females on day 8 and treated with DMSO, JH and ACPD+JH, respectively. Arrow heads indicate patency. Scale bars: 5 μm. (F) Immunoprecipitation (IP) and Western blotting (WB) showing inhibition of ACPD on JH-induced disassociation of p-Par3 and β-Cat. (G) Distribution of β-Cat in the follicular epithelium treated with DMSO, JH and ACPD+JH. Arrow heads indicate patency. Scale bars: 5 μm.

Par3 phosphorylation leads to its conformational change and release from E-Cad binding [32,36,43]. Knowing that aPKC and Par3 were required for patency opening (Fig 2B), we then performed Western blot to examine the phosphorylation status of aPKC and Par3 in ovarian follicular epithelia of adult females during the first gonadotropic cycle. As shown in Figs 3A and S2A, phosphorylation of aPKC (p-aPKC) and Par3 (p-Par3) was observed at 4 days PAE and significantly enhanced on day 6–8, which coincided with elevated JH titer and patency development (Fig 1A). We next defined the effect of JH on aPKC and Par3 phosphorylation using the cultured follicular epithelium. As displayed in Figs 3B and S2B, the levels of p-aPKC and p-Par3 significantly increased at 15–30 min post JH application. Administration of an aPKC-specific inhibitor, 2-acetyl-1, 3-cyclopentanedione (ACPD) [44] prior to JH treatment precluded JH-induced phosphorylation of aPKC and Par3 (Figs 3C and S2C). The data suggest that aPKC mediates JH-induced phosphorylation of Par3. We next performed immunostaining with anti-phospho-aPKC and anti-phospho-Par3 antibodies to visualize the subcellular localization of p-aPKC and p-Par3 in the follicular epithelium of vitellogenic females on day 8. As shown in Fig 3D and 3E, p-aPKC and p-Par3 primarily localized at tricellular patency but not bicellular junctions in cultured follicular epithelia treated with DMSO, suggesting the requirement of aPKC and Par3 phosphorylation in ZA disassembly and patency opening. Additional JH treatment led to extended distribution of p-aPKC and p-Par3 along with enlarged patency (Fig 3D and 3E). Prior application of ACPD restrained JH-induced extension of p-aPKC and p-Par3 distribution and enlargement of tricellular patency (Fig 3D and 3E). Immunoprecipitation and Western blot displayed that Par3 but not p-Par3 interacted with β-Cat (Fig 3F). Immunostaining exhibited that JH treatment led to diminished β-Cat distribution at bicellular junctions along with enlarged opening of patency (Fig 3G). Pretreatment of ACPD resulted in extended distribution of β-Cat at bicellular junctions as well as shrunk patency (Fig 3G). Collectively, the data indicate that JH triggers Par3 phosphorylation via aPKC for ZA disassembly and patency induction.

**Fig 4:**
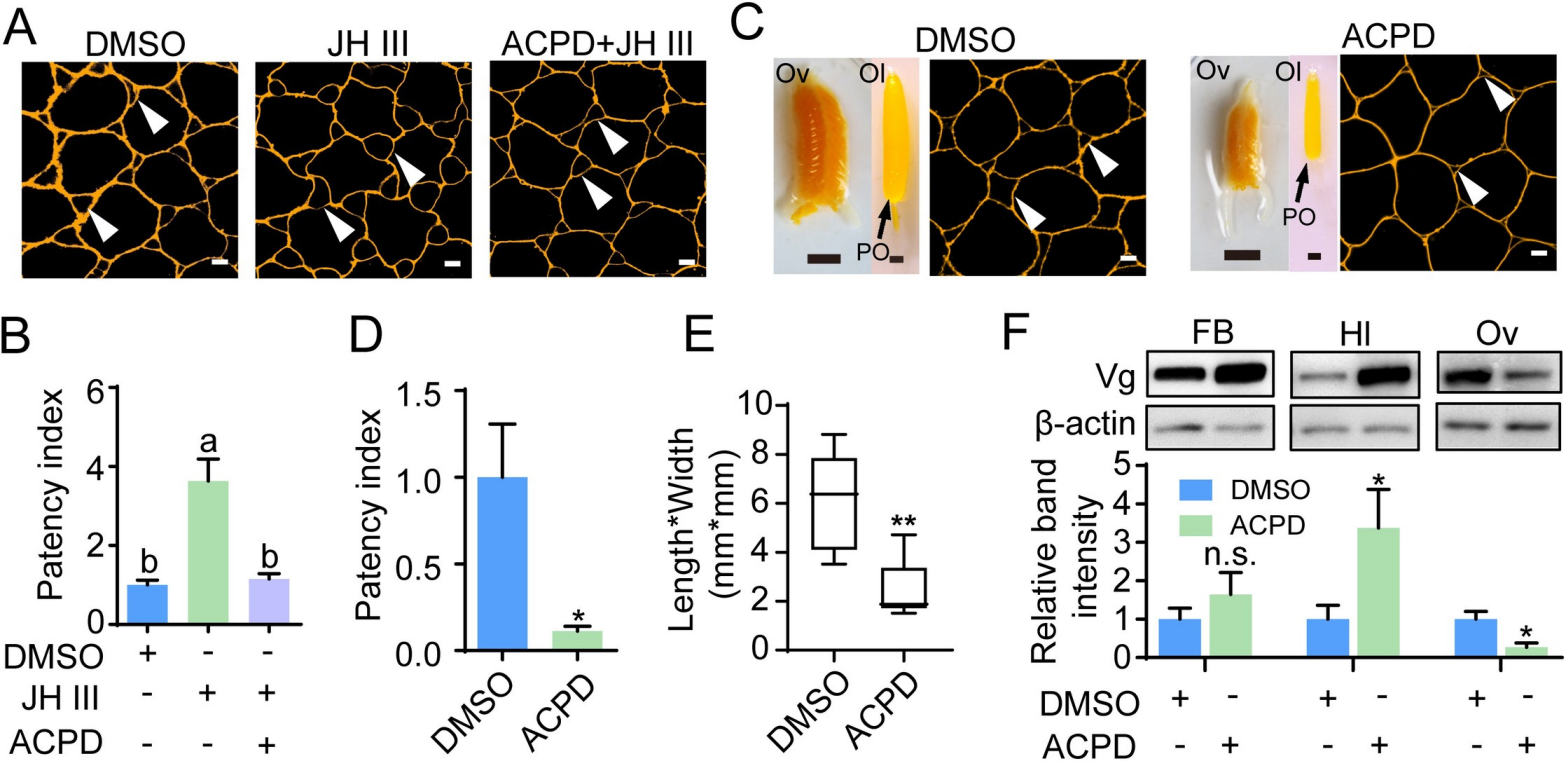
Effect of ACPD treatment on JH-induced patency and vitellogenin transport. (A) Effect of ACPD treatment on JH-induced patency in cultured follicular epithelium isolated from adult females on day 7. Arrow heads indicate patency. Scale bars: 5 μm. (B) Statistical analysis of patency index in cultured follicular epithelium as represented by Fig 4A. Means labeled with different letters indicate significant difference at *P* < 0.05. n = 8–10. (C) Effect of ACPD on patency, oocyte maturation and ovarian growth of 7-day-old adult females. Vitellogenic adult females were injected with 40 μg ACPD once per day from 5 to 7 days PAE and phenotypes were evaluated 6 h after the final injection. Ov, ovary; Ol, ovariole; Po, primary oocyte. Arrow heads indicate patency. Scale bars: ovary, 5 mm; ovariole, 0.5 mm; follicular epithelium, 5 μm. (D) Statistical analysis of patency index in the follicular epithelium of 7-day-old adult females subjected ACPD injection vs. the DMSO control. *, *P* < 0.05. n = 8–10. (E) Statistical analysis for the index of primary oocytes of ACPD- and DMSO-treated adult females at 7 days PAE. **, *P* < 0.01 when compared to the DMSO control. n = 8–10. (F) Western blot showing the abundance (upper panel) and quantitative analysis of band intensity (lower panel) of vitellogenin proteins in the fat body (FB), hemolymph (Hl) and ovary (Ov) of ACPD*-*injected adult females vs. the DMSO controls. * *P* < 0.05 compared to the respective DMSO control. n = 8–10. n.s., no significant difference.

To further characterize the role of p-aPKC and p-Par3 in JH-induced patency, we performed both in vivo and in vitro studies with ACPD treatment. In cultured follicular epithelia of vitellogenic females on day 7, preincubation of ACPD inhibited JH-induced patency dilatation (Fig 4A). Statistically, the average patency index of JH-treated follicular epithelia increased 3.6 folds compared to that of DMSO controls (Fig 4B). In the follicular epithelia treated with ACPD and JH, the average patency index reduced to 1.2 (Fig 4B). Injection of ACPD into vitellogenic females also abolished patency induction (Fig 4C). The average patency index decreased by 88.7% in the follicular epithelium of ACPD-injected females compared to the DMSO control (Fig 4D). As a consequence, the primary oocytes and ovaries of ACPD-injected adult females were much smaller than the DMSO control at 7 days PAE (Fig 4C). In comparison with that of DMSO controls, the average length*width index of ACPD-treated primary oocytes reduced by 58.9% (Fig 4E). Notably, while remaining constant in the fat body, Vg protein levels increased 3.4 folds in the hemolymph but declined by 73.2% in the ovary of 7-day-old adult females injected with ACPD compared to the DMSO control (Fig 4E). The data indicate that ACPD treatment causes Vg accumulation in the hemolymph due to blocked patency opening and that phosphorylation of aPKC and Par3 is required for JH-dependent ZA remodeling and patency induction.

### JH induces aPKC and Par3 phosphorylation via the GPCR-Cdc42 signaling cascade

**Fig 5:**
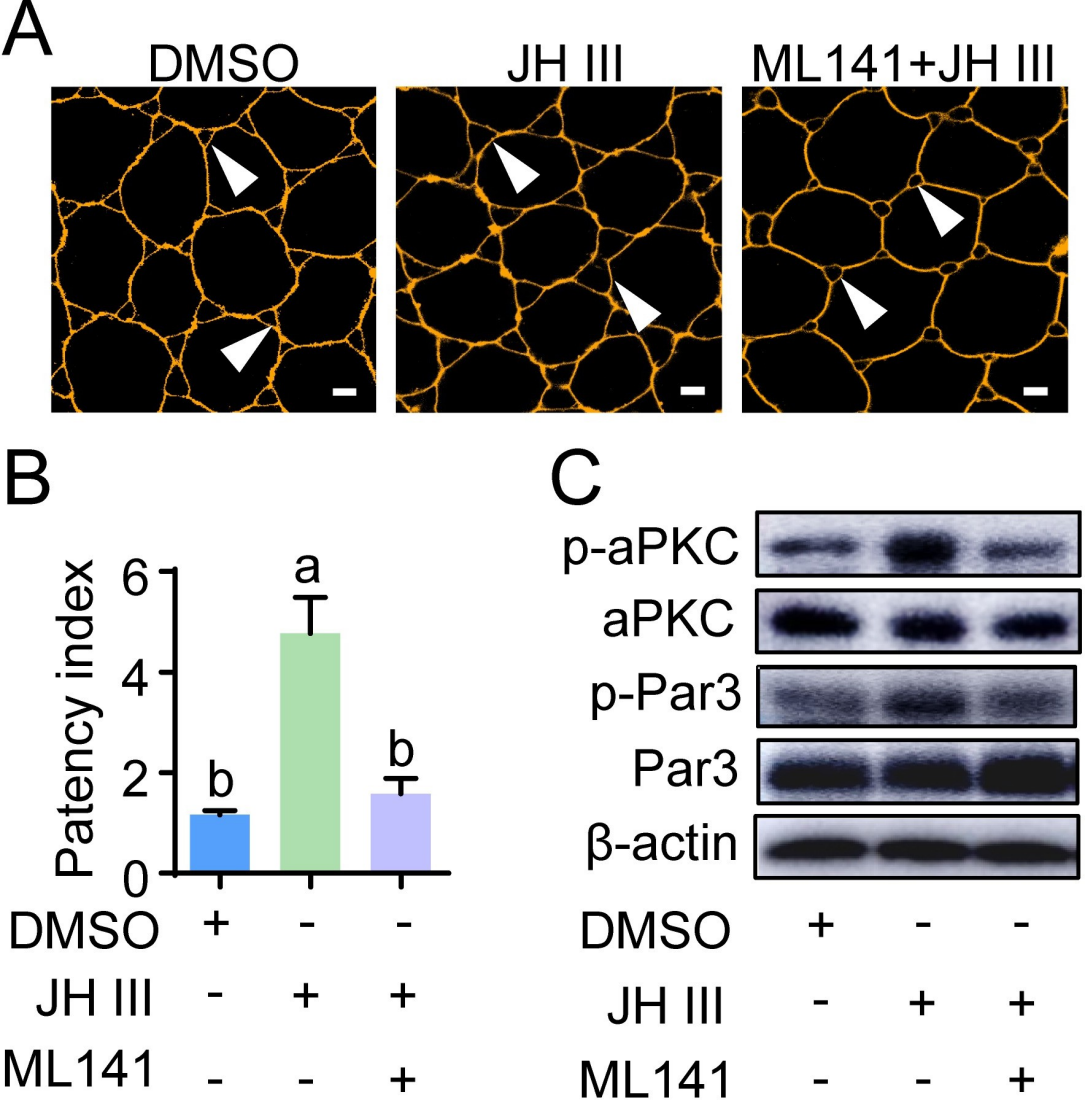
Involvement of Cdc42 in JH-dependent patency and phosphorylation of aPKC and Par3. (A) Inhibitory role of a Cdc42 specific inhibitor, ML141 (at 10 μM) in JH-induced patency of cultured follicular epithelium from 7-day-old adult females. Arrow heads indicate patency. Scale bars: 5 μm. (B) Statistical analysis of patency index in cultured follicular epithelia treated with JH, ML41+JH and DMSO. Means labeled with different letters indicate significant difference at *P* < 0.05. n = 8–10. (C) Effect of ML141 treatment on JH-stimulated phosphorylation of aPKC and Par3.

It has been previously reported that Cdc42 interacts with Par6, thereby activating aPKC for Par3 phosphorylation [45,46]. We evaluated the role of Cdc42 in JH-stimulated aPKC activation, Par3 phosphorylation and patency initiation using the cultured follicular epithelia of 7-day-old adult females. Prior application of Cdc42 inhibitor, ML141 blocked JH-induced patency (Fig 5A). The patency index dropped 3-fold in follicular epithelia pretreated with ML141 followed by JH application compared to that of JH treatment alone (Fig 5B). The levels of p-aPKC and p-Par3 significantly declined in ML141-preincubated follicular epithelia further treated with JH (Figs 5C and S3). The data imply that JH promotes aPKC and Par3 phosphorylation via Cdc42.

**Fig 6:**
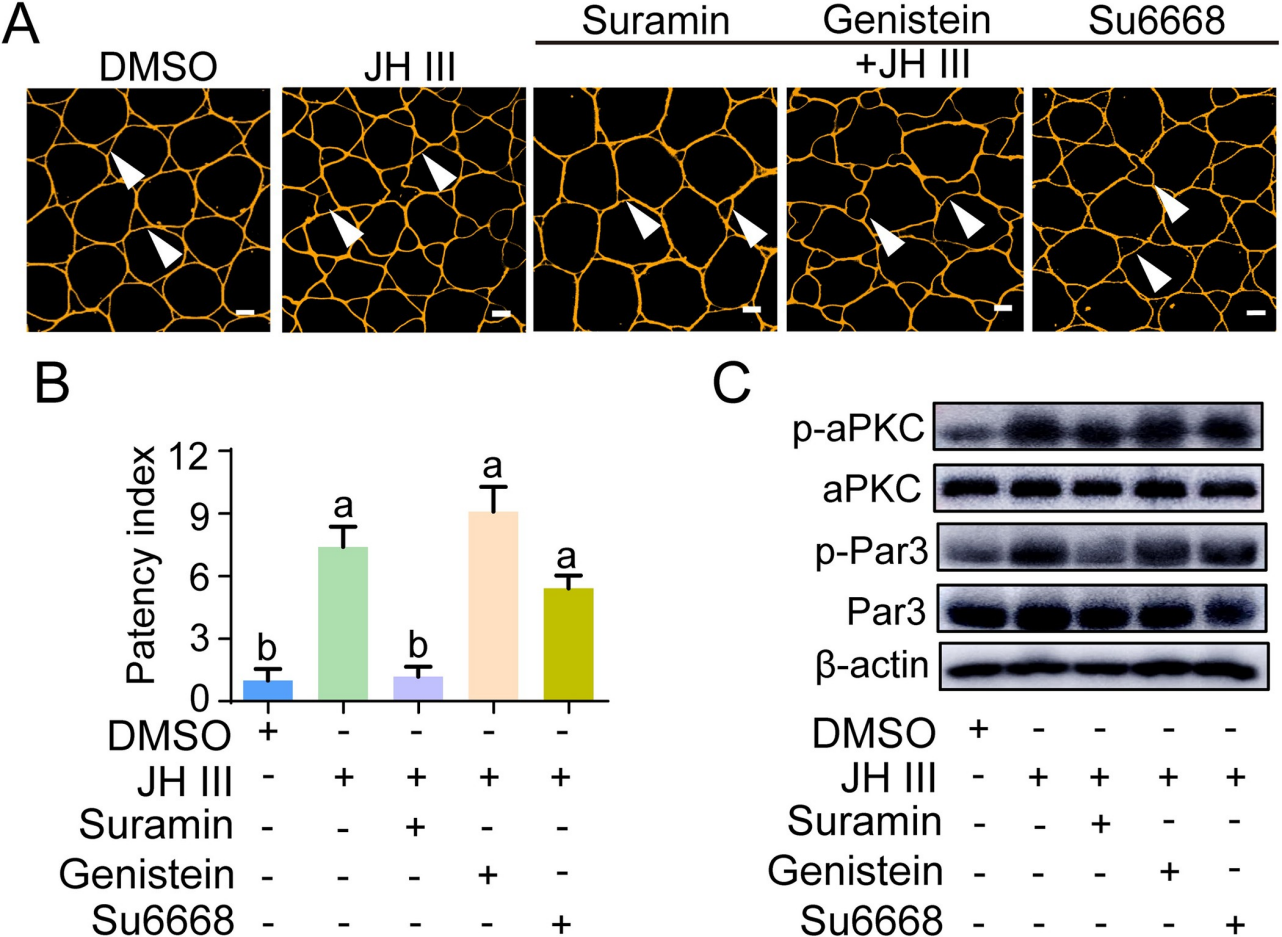
The role of GPCR in patency opening and phosphorylation of aPKC and Par3. (A) Effect of GPCR inhibitor suramin (1 μM) as well as RTK inhibitors genistein (10 μM) and Su6668 (10 μM) on JH-induced patency of cultured follicular epithelium from 7-day-old adult females. Arrow heads indicate patency. Scale bars: 5 μm. (B) Statistical analysis of patency index in cultured follicular epithelia treated with DMSO, JH, suramin, genistein and Su6668. Means labeled with different letters indicate significant difference at *P* < 0.05. n = 8–10. (C) Effect of suramin, genistein and Su6668 treatment on JH-stimulated phosphorylation of aPKC and Par3.

As GPCR and RTK convey JH membrane signaling [2,10,13,18,47], we next applied their inhibitors in cultured follicular epithelia from adult females on day 7. When follicular epithelia treated with GPCR inhibitor suramin plus JH, the extent of patency became significantly smaller than that of JH treatment alone (Fig 6A and 6B). However, when RTK inhibitors genistein and Su6668 were applied, patency size was insignificantly altered (Fig 6A and 6B). Western blot demonstrated that pretreatment of suramin but not genistein or Su6668 caused significant decrease of p-aPKC and p-Par3 levels (Figs 6C and S4). The data indicate that GPCR is involved in JH-stimulated phosphorylation of aPKC and Par3 as well as ZA disassembly and patency enlargement.

## Discussion

Ovarian follicle cells are organized as a monolayer via intercellular junctions and function as a barrier for the enveloped oocytes [2,18]. During insect vitellogenesis and oogenesis, JH-induced patency in the follicular epithelium is prerequisite for hemolymph Vg to gain access to oocyte membrane where Vg is subsequently taken up by maturing oocytes via VgR-mediated endocytosis [18,48]. By imaging the distribution of β-Cat, we analyzed ZA dynamics in the follicular epithelium during the first gonadotropic cycle of *L*. *migratoria*. In previtellogenic phase, β-Cat was present in both bicellular junctions and tricellular vertices. However, at early vitellogenic stage when patency was initiated, β-Cat started disappearing from tricellular vertices. While tricellular patency enlarged in mid- and late-vitellogenic follicular epithelia, β-Cat was restricted to bicellular contacts. These observations support the previous reports on the involvement of ZA remodeling in patency opening in vitellogenic follicle epithelium of *L*. *migratoria*, *R*. *Prolixus* and *D*. *melanogaste*r [25–27,30]. dsRNA-mediated knockdown of *β-Cat* caused removal of cell adhesions and loss of patency. Consequently, oocyte maturation and ovarian growth arrested. Such defective phenotypes were also seen with depletion of *aPKC*, *Par6* and *Par3*. These data provide a clear indication that ZA remodeling is involved in JH-induced patency and vitellogenesis. Previously, we reported that JH induces patency via activating Na^+^/K^+^-ATPase and follicular cell shrinkage [24]. We found in this study that while incubation of JH with cultured follicular epithelia caused patency enlargement, the distribution of β-Cat limited to shortened bicellular junctions. It is conceivable that in addition to Na^+^/K^+^-ATPase-modulated cell shrinkage, JH promotes ZA disassembly for patency opening, allowing paracellular transport of yolk protein precursors to cross follicular epithelium barrier and reach oocyte surface during vitellogenesis.

Patency opening occurs at onset of vitellogenesis and correlates with increased JH titers [1–3,5]. We observed in the present study that JH promoted aPKC and Par3 phosphorylation, whereas pretreatment of aPKC inhibitor ACPD abolished JH-induced phosphorylation of aPKC and Par3. Thus, it is plausible that aPKC mediates JH-induced Par3 phosphorylation. Interestingly, phosphorylated aPKC localized around tricellular patency but not bicellular junctions. aPKC interacts with Par6 through their PB1 domains and binds to Par3 at the conserved region 3 (CR3) through its kinase domain, which forms a stable Par complex [49,50]. Our data demonstrated that along with JH-induced patency opening, Cdc42-mediated phosphorylation of aPKC led to Par3 phosphorylation and its subsequent dissociation from β-Cat. While β-Cat was distributed at bicellular junctions, p-aPKC together with p-Par3 localized at tricellular patency. Additional JH treatment resulted in more distribution of p-aPKC at tricellular patency but less distribution of β-Cat at bicellular contacts, together with enlarged patency. This is in agreement with previous reports that aPKC-mediated Par3 phosphorylation led to its dissociation from β-Cat [32, 40]. Our findings extend the view on aPKC and Par3 phosphorylation by JH regulation. aPKC-mediated Par3 phosphorylation in the remodeling of occluding junctions has been reported in paracellular transport crossing other types of epithelia [51,52]. Our results provide new insight into JH regulation of ZA remodeling in the follicular epithelium for patency opening. Cumulative studies have established that the evolutionarily conserved Par6/aPKC/Par3 complex plays a crucial role in apical-basal polarity of epithelial cells [32,39,53]. In addition, the Scribble (Scrib) complex including Scrib, Discslarge (Dlg) and Lethal giant larvae (Lgl) as well as the Hippo pathway coordinate with the Par6/aPKC/Par3 modules in regulation of cell polarity [54,55]. The establishment and maintenance of cell polarity are essential for multiple cellular processes including cell adhesion, cell shape and paracellular trafficking. Our data, together with the published studies, suggest a link between patency and cell polarity. By utilizing a series of inhibitors, we further unfolded the involvement of GPCR and Cdc42 in JH-stimulated phosphorylation of aPKC and Par3 along with ZA remodeling and patency enlargement. The above data shed light on a molecular mechanism by which JH triggers Par3 phosphorylation via the GPCR-Cdc42-Par6-aPKC signaling cascade, which in turn induces ZA disassembly at tricellular vertices for patency opening. It is noteworthy that β-Cat was used as a marker to analyze ZA disassembly in the present study. The effect of Par3 phosphorylation on ZA disassembly was observed by imaging the subcellular distribution of p-Par3 and β-Cat, along with extrapolation from published studies [27,43,56]. Our immunoprecipitation and Western blot demonstrated that Par3 phosphorylation led to its dissociation from β-Cat. Immunostaining showed that β-Cat localized at bicellular junctions when patency enlarged. In contrast, p-Par3 localized at tricellular patency but not bicellular junctions. Recent studies have demonstrated that GPCR conveys JH signaling in insect metamorphosis and reproduction. In the cotton bollworm *Helicoverpa armigera*, JH promotes Broad-complex phosphorylation via the context of GPCR-PLC-PKC pathway, thereby repressing ecdysone-induced metamorphosis [47]. In *L*. *migratoria*, JH activates the GPCR-PLC-IP3R-PKC signaling cascade that triggers Na^+^/K^+^-ATPase phosphorylation for patency induction and Vg transport [24]. JH also promotes VgR phosphorylation via the GPCR-PLC-PKCι cascade for receptor-mediate endocytosis and Vg deposition in oocytes [17]. It is of interest to identify the specific GPCR and other molecules involved in the regulation of JH-stimulated ZA remodeling and patency opening.

**Fig 7:**
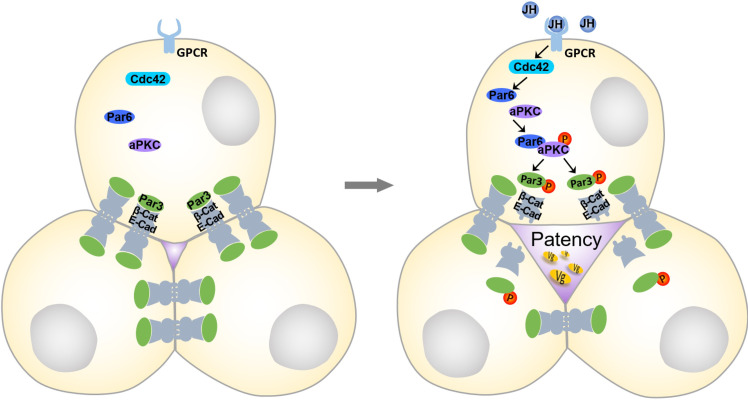
A proposed model for JH-stimulated ZA disassembly and vitellogenin transport in the follicular epithelium. JH promotes Par3 phosphorylation via a signaling cascade including GPCR, Cdc42, Par6 and aPKC. Phosphorylated Par3 disassociates from E-Cad/β-Cat complex, consequently causing ZA disassembly at tricellular vertices and patency opening. Patency allows yolk protein precursors to pass through the barrier of follicular epithelium and reach the surface of maturing oocytes.

In synopsis with our findings and current knowledge, we propose a model for the regulation of ZA remodeling and patency opening during JH-stimulated vitellogenesis and oogenesis (Fig 7). JH activates a signaling cascade including GPCR, Cdc42, Par6 and aPKC, which triggers Par3 phosphorylation. Phosphorylated Par3 disassociates from E-Cad, causing zonula adherens disassembly at tricellular contacts in the follicular epithelium. Separation of adhesion junctions at tricellular vertices subsequently leads to enlarged opening of patency for Vg transport.

## Materials and methods

### Insects

The gregarious colony of migratory locusts was maintained under 14L:10D photoperiod and at 30±2°C as previously described [57]. ACPD (Sigma-Aldrich) was intra-abdominally injected at 40 μg once per day from 5 to 7 days PAE. The phenotypes were evaluated at 6 h after the final injection. Adult females injected with the same volume of DMSO were used as controls.

### *In vitro* patency induction

Ovarioles were dissected from ovaries of 7-day-old adult females and desheathed in medium containing 60% Schneider’s *Drosophila* medium and 40% Basic Medium Eagle as previously described [58], followed by incubation with fresh medium containing 0.1 μM JH III (Santa Cruz) for 1 h. In pharmacological experiments with inhibitors, ovarioles were separately exposed to ACPD (0.1 μM), ML141 (10 μM, MedChemExpress), suramin (1 μM, Sigma-Aldrich), genistein (10 μM, MedChemExpress) and Su6668 (10 μM, MedChemExpress) for 30 min, followed by JH III (Santa Cruz) treatment for additional 1 h. DMSO was used as the control. After the incubation, CellMask Orange Plasma Membrane Stain (Invitrogen) was added and further incubated for 5 min. The images were captured with a ZEISS LSM710 laser confocal microscope and processed with Zen2012 software (Carl Zeiss). Area of follicle cell and patency were measured with Image-Pro Plus 6.0 (NIH). Patency index was calculated by comparing the area of patency surrounding a follicle cell to the area of this follicle cell.

### RNA interference and tissue imaging

Double-stranded RNA (dsRNA) was synthesized using the T7 RiboMAX Express System (Promega) according to the manufacturer’s manual. Adult females were intra-abdominally injected with 15 μg dsRNA (3μg/μl H_2_O) within 12 h after eclosion and boosted once on day 5. Phenotypes were examined at 8 days PAE. Green fluorescent protein (GFP) dsRNA was used as a mock control. Primers used in the dsRNA synthesis are listed in S1 Table. Ovaries and ovarioles were imaged with Canon EOS550D camera and M205A microscope (Leica), respectively.

### RNA isolation and qRT-PCR

Total RNA was isolated from the ovary of 8-day-old adult females using Trizol reagent (Invitrogen). First-strand cDNA was reverse transcribed with FastQuant RT Kit (with gDNase) (Tiangen). qRT-PCR was performed using a Light cycler 96 (Roche) and a SuperReal Premix Plus kit (Tiangen), initiated at 95°C for 15 min, followed by 40 cycles of 95°C for 10 s, 60°C for 20 s and 72°C for 30 s. Melting curve analysis was performed to confirm the specificity of amplification. Ribosomal protein 49 (rp49) was used as a reference control. The 2^-ΔΔCt^ method was applied to calculate relative expression levels. Primers used for qRT-PCR are listed in S1 Table.

### Immunostaining

Sheath-free ovarioles were fixed in 4% paraformaldehyde and then permeabilized in 0.3% Triton X-100. F-actin and nuclei were stained with 0.165 μM Phalloidin-Alexa Flour 488 (Invitrogen) and 5μM Hoechst 33342 (Sigma-Aldrich), respectively. β-Cat and phosphorylated aPKC staining was conducted with primary antibodies against β-Cat (Cell Signaling Technology) and phospho-aPKC (Thr555/563) (Invitrogen) and corresponding Alexa Fluor 594 goat anti-rabbit IgG secondary antibody (Invitrogen). Images were captured and processed as described above.

### Antibody preparation

cDNA fragments coding for locust Par3, aPKC and Par6 were amplified with the respective primers (S1 Table) using *Pfu* DNA polymerase (Novoprotein), cloned into pET-32a (+)-His, and confirmed by sequencing. The recombinant peptides were expressed in Rosetta host cells under IPTG induction, purified by Ni^2+^-His affinity column and examined by SDS-PAGE. Polyclonal antibodies were raised in New Zealand White rabbits using the purified peptides mixed with Freund’s complete adjuvant (Sigma-Aldrich) to form a stable emulsion for immunization. The rabbits were injected subcutaneously at four sites, and boosted once a week for a total of 4 times. The antiserum specificity was verified by Western blot using proteins extracted from ovaries of adult females subjected to respective gene knockdown. The custom antibody against p-Par3 was generated by Beijing Protein Innovation. Its specificity was verified by Western blot using protein extracts isolated from follicular epithelia of adult females subjected to *Par3* RNAi, *aPKC* RNAi and phosphatase λpp treatment (S5 Fig).

### Western blot and immunoprecipitation

Total proteins were isolated from the tissues of adult females, and quantified with a BCA protein assay kit (Applygen Technologies) as described previously [59]. Western blots were performed using the primary antibodies against β-Cat, Par3, aPKC, Par6, p-Par3, p-aPKC (Invitrogen), and VgA [60], the corresponding HRP-conjugated secondary antibodies (CWBIO) and an enhanced chemiluminescent reagent (CWBIO). Application of β-actin was used as the loading control. Bands were visualized by an Amersham Biosciences Imager 600 (GE Healthcare) and quantified by ImageJ. For immunoprecipitation, protein extracts were precleared with Protein A-agarose (Sigma-Aldrich) for 1 h at 4°C and incubated with the antibody of β-Cat at 4°C overnight. The immunocomplexes were captured with Protein A agarose (Sigma-Aldrich) and eluted in Laemmli sample buffer, followed by Western blot with antibodies.

### Data analysis

Statistical analysis was performed with Student’s *t*-test or one-way analysis of variance (ANOVA) with Tukey’s post-hoc test using STATISTICA 12.0 software (StatSoft). Significant difference was considered at *P* < 0.05. Values were reported as mean ± SE.

## Supporting information

S1 Fig Quantitative analysis of β-Cat, Par3, aPKC and Par6 band intensity from Western blots representatively shown in Fig 2A. *, *P* < 0.05 compared to the respective dsGFP control. n = 3.
(TIF)

S2 Fig Quantitative analysis of band intensity from Western blots representatively shown in Fig 3. (A), (B) and (C) Relative band intensity of p-Par3 and p-aPKC from Western blots representatively shown in Fig 3A, 3B and 3C, respectively. *, *P* < 0.05 compared to the DMSO control; n.s., no significant difference compared to the DMSO control; means labeled with different letters indicate significant difference at *P* < 0.05. n = 3.
(TIF)

S3 Fig Quantitative analysis of p-Par3 and p-aPKC band intensity from Western blots representatively shown in Fig 5C. Means labeled with different letters indicate significant difference at *P* < 0.05. n = 3.
(TIF)

S4 Fig Quantitative analysis of p-Par3 and p-aPKC band intensity from Western blots representatively shown in Fig 6C. Means labeled with different letters indicate significant difference at *P* < 0.05. n = 3.
(TIF)

S5 Fig Validation of p-Par3 antibody specificity. Upper panel: Western blots using protein extracts isolated from follicular epithelia of 8-day-old adult females subjected to *Par3* RNAi (A), *aPKC* RNAi (B) and λPP treatment (C). Lower panel: quantitative analysis of band intensity from Western blots representatively shown in the upper panel. *, *P* < 0.05; **, *P* < 0.01; compared to the dsGFP control. n = 3.
(TIF)

S1 Table Primers used for PCR cloning, RNAi and qRT-PCR. (DOCX)
