# Supplementary figures and images for: Juvenile hormone promotes paracellular transport of yolk proteins via remodeling zonula adherens at tricellular junctions in the follicular epithelium
Source: PLoS Genet. 2022 Jun 27;18(6):e1010292. doi: 10.1371/journal.pgen.1010292 (PMC9269875; doi:10.1371/journal.pgen.1010292)

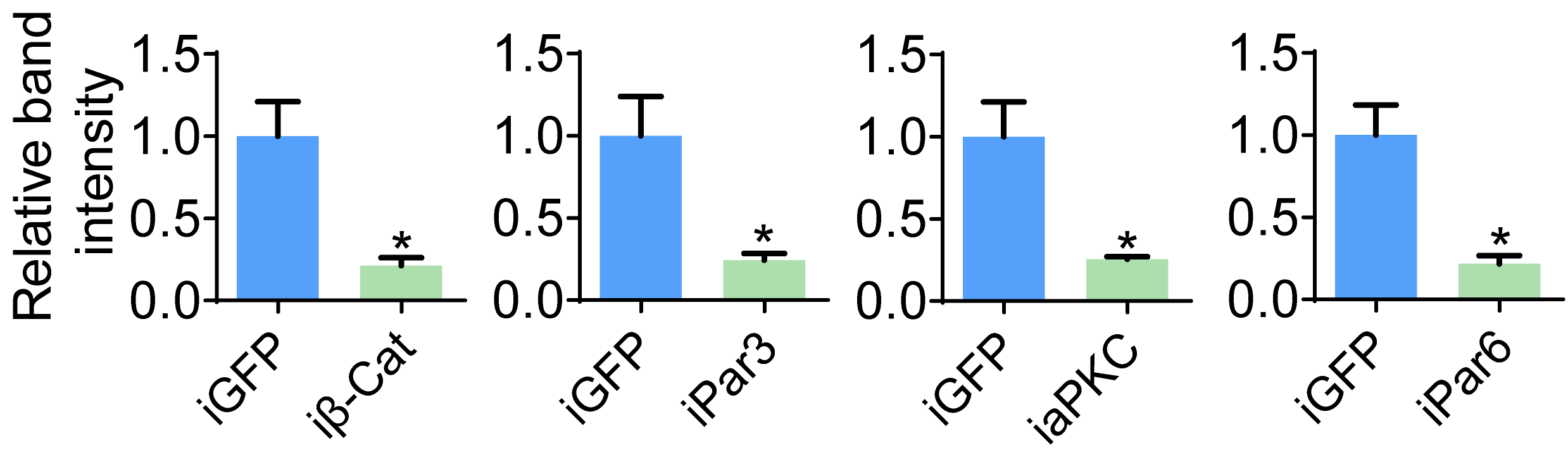

Supplement: S1 Fig — *, P < 0.05 compared to the respective dsGFP control. n = 3. (TIF) [file pgen.1010292.s001.tif]

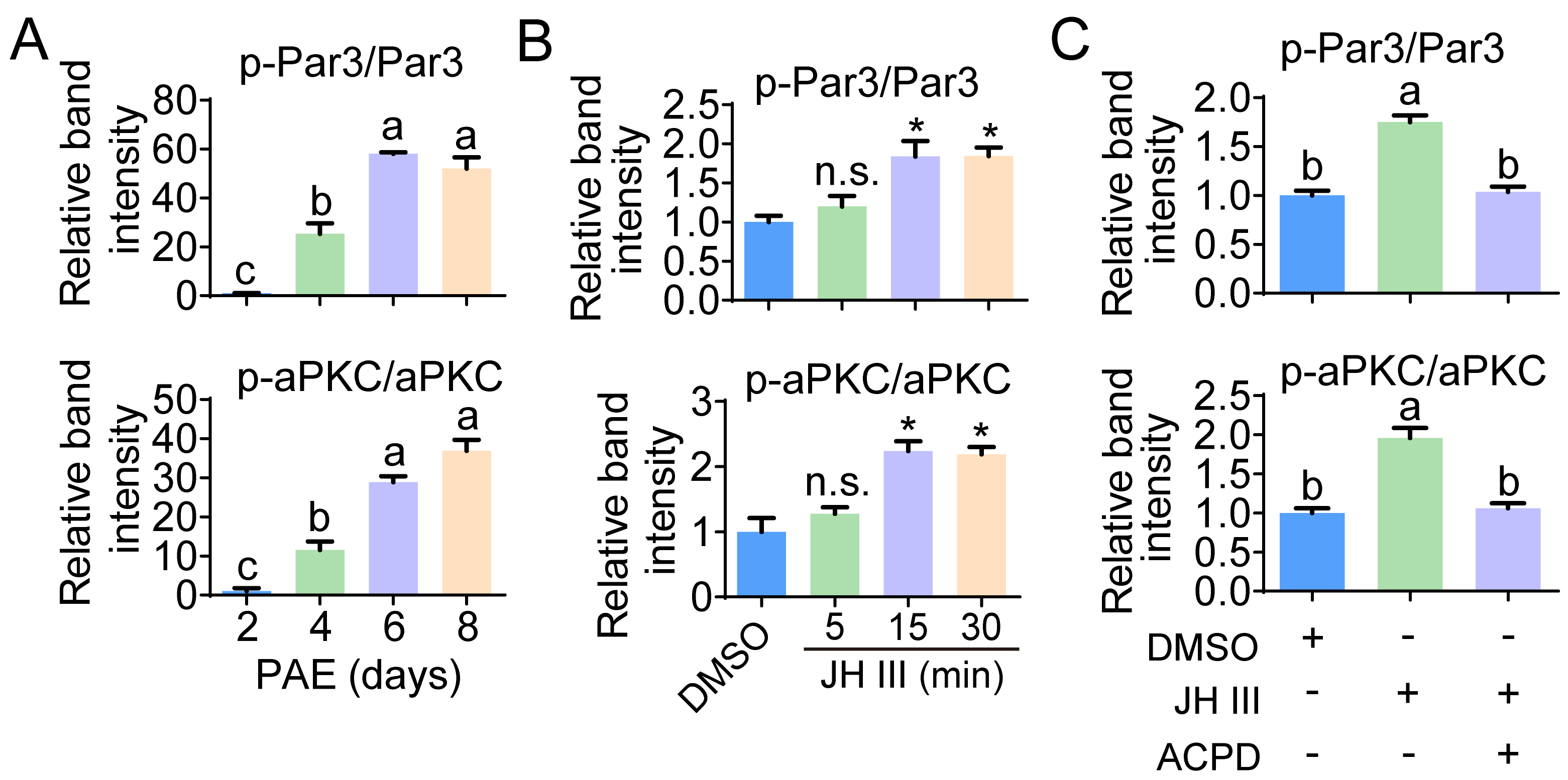

Supplement: S2 Fig — (A), (B) and (C) Relative band intensity of p-Par3 and p-aPKC from Western blots representatively shown in Fig 3A, 3B and 3C, respectively. *, P < 0.05 compared to the DMSO control; n.s., no significant difference compared to the DMSO control; means labeled with different letters indicate significant difference at P < 0.05. n = 3. (TIF) [file pgen.1010292.s002.tif]

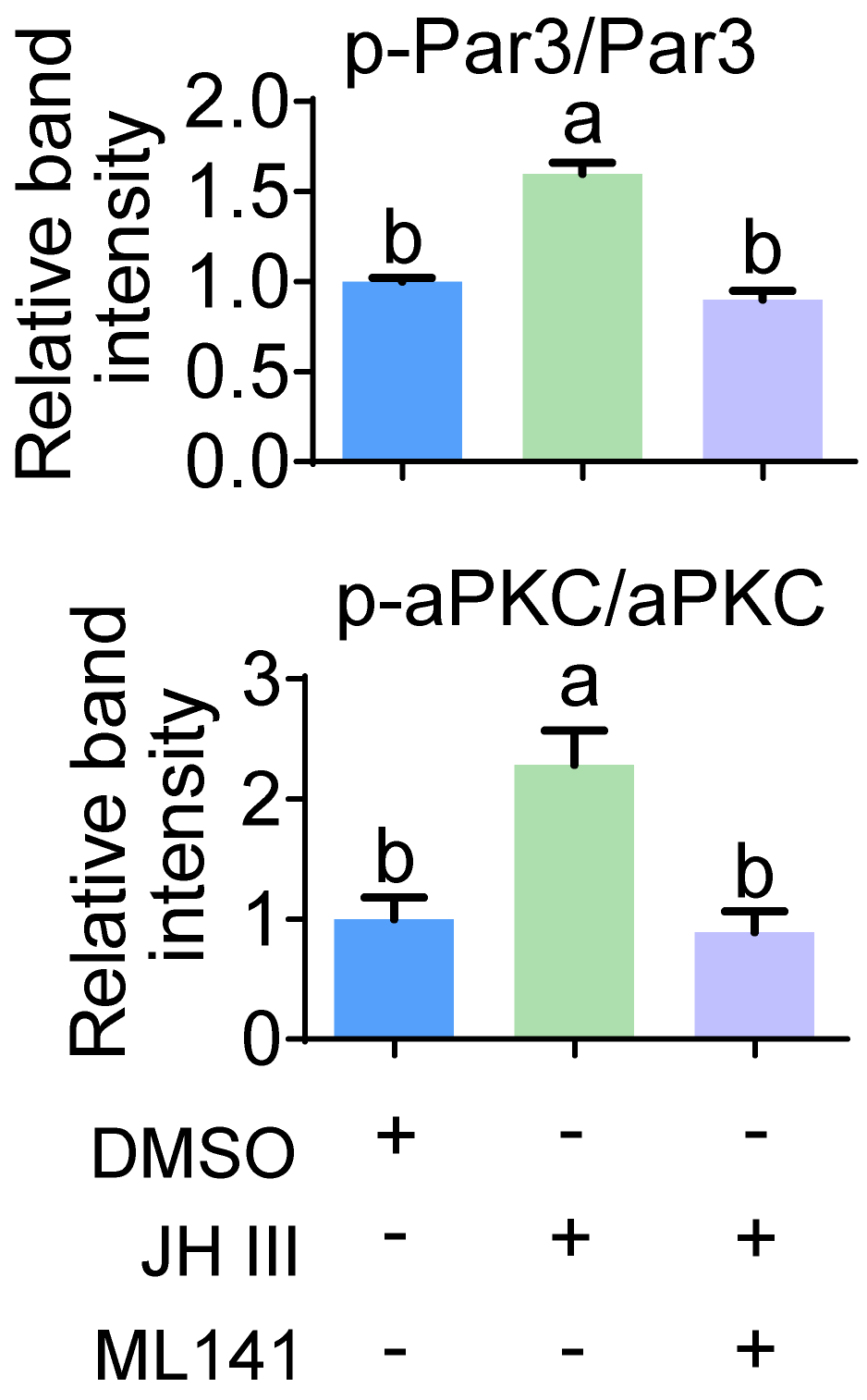

Supplement: S3 Fig — Means labeled with different letters indicate significant difference at P < 0.05. n = 3. (TIF) [file pgen.1010292.s003.tif]

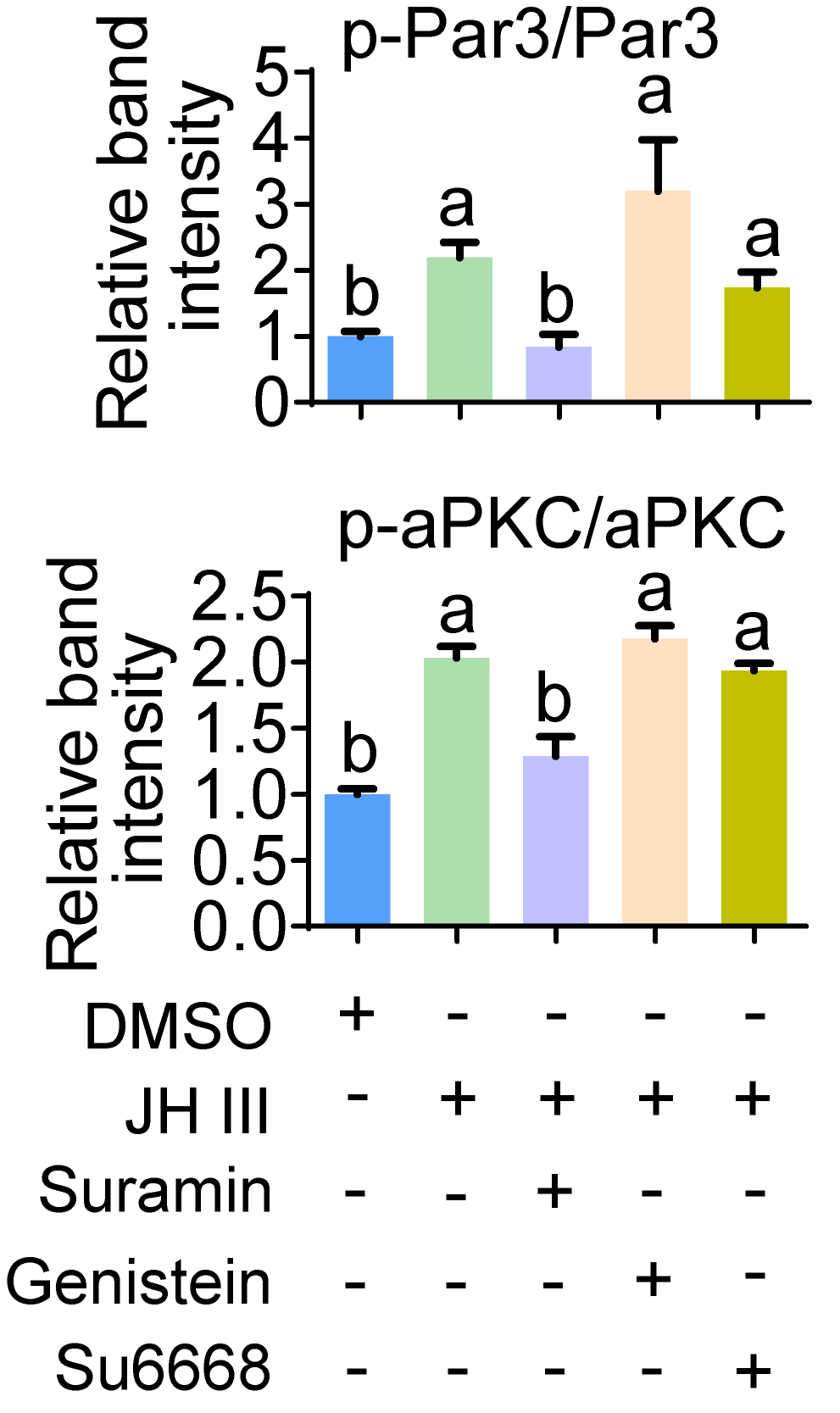

Supplement: S4 Fig — Means labeled with different letters indicate significant difference at P < 0.05. n = 3. (TIF) [file pgen.1010292.s004.tif]

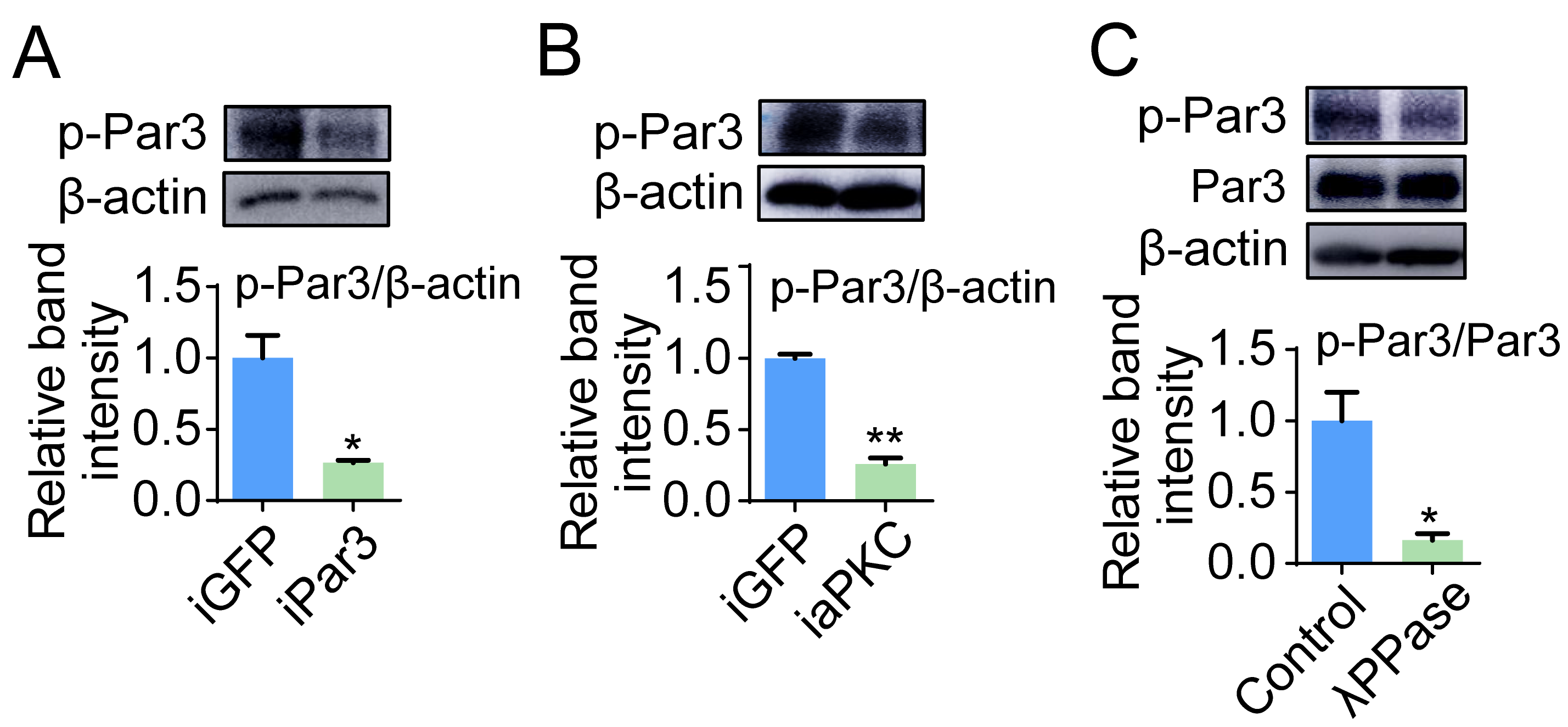

Supplement: S5 Fig — Upper panel: Western blots using protein extracts isolated from follicular epithelia of 8-day-old adult females subjected to Par3 RNAi (A), aPKC RNAi (B) and λPP treatment (C). Lower panel: quantitative analysis of band intensity from Western blots representatively shown in the upper panel. *, P < 0.05; **, P < 0.01; compared to the dsGFP control. n = 3. (TIF) [file pgen.1010292.s005.tif]
