# Supplementary material for: Juvenile hormone promotes paracellular transport of yolk proteins via remodeling zonula adherens at tricellular junctions in the follicular epithelium
Source: PLoS Genet. 2022 Jun 27;18(6):e1010292. doi: 10.1371/journal.pgen.1010292 (PMC9269875; doi:10.1371/journal.pgen.1010292)
Supplement: S1 Table — (DOCX) [file pgen.1010292.s006.docx]

S1 Table. Primers used for PCR cloning, RNAi and qRT-PCR.

|  | **Gene** | **Forward primer (5’ to 3’)** | **Reverse primer (5’ to 3’)** |
| --- | --- | --- | --- |
| **Prokaryotic expression** | *Par3** | CGGGGTACCAAGGATACAGAACGGAGG | CCGGAATTCGTCCTCCTGCATTTGGAT |
|  | *aPKC** | CGGGGTACCAGAAGTCCGTTCGATATT | CCCAAGCTTCACGCAGTCCTCCAGCGA |
|  | *Par6** | CCGGAATTCGCGTGAGATGAATATTCC | CGGGGTACCCTTGTACCAGGTGGTCTT |
| **RNAi** | *β-cat** | AGTTGCTGCTGGAGTTCTCTG | GCATTGGATACTGGCGACCT |
|  | *Par3* | GCAACAGCAGCAGCAGAAC | CAGCCAGTGGTGCCTCATAG |
|  | *aPKC* | TTGCCAAGGAGAAGACAGGAG | CACGACCAATAACACGGATCAG |
|  | *Par6* | ATTCATCTCACGCCTTGTACCA | GCCACCTGTCATATCCACGAT |
|  | *GFP* | CACAAGTTCAGCGTGTCCG | GTTCACCTTGATGCCGTTC |
| **qRT-PCR** | *β-cat* | GTCAGTCCATAGCCAGCAGTG | GGCTCCAACAGTTCCTTCAACA |
|  | *Par3* | GAGGCACCACTGGCTGATGT | AACACCGCAGTCTGAGTCTCC |
|  | *aPKC* | CGTGGTGGTGACCTGATGT | GGAAGTTGAGTGCAAGACTGAT |
|  | *Par6* | CTCCCAGTAAACCCAAGACACA | CAGCCTCACTCTGCGATATGT |
|  | *Rp49* | CGTAAACCGAAGGGAATTGA | GAAGAAACTGCATGGGCAAT |

* GenBank: Par3, MT036384; aPKC, MT036383; Par6, MT036385; β-Cat, MT036387.
